# Identification of the Molecular Site of Ivabradine Binding to HCN4 Channels

**DOI:** 10.1371/journal.pone.0053132

**Published:** 2013-01-04

**Authors:** Annalisa Bucchi, Mirko Baruscotti, Marco Nardini, Andrea Barbuti, Stefano Micheloni, Martino Bolognesi, Dario DiFrancesco

**Affiliations:** 1 The PaceLab, Department of Life Sciences, Università degli Studi di Milano, Milano, Italy; 2 Centro Interuniversitario di Medicina Molecolare e Biofisica Applicata, Università degli Studi di Milano, Milano, Italy; 3 Laboratory of Protein Biochemistry, Department of Life Sciences, Università degli Studi di Milano, Milano, Italy; Virginia Commonwealth University, United States of America

## Abstract

Ivabradine is a specific heart rate-reducing agent approved as a treatment of chronic stable angina. Its mode of action involves a selective and specific block of HCN channels, the molecular components of sinoatrial "funny" (f)-channels. Different studies suggest that the binding site of ivabradine is located in the inner vestibule of HCN channels, but the molecular details of ivabradine binding are unknown. We thus sought to investigate by mutagenesis and *in silico* analysis which residues of the HCN4 channel, the HCN isoform expressed in the sinoatrial node, are involved in the binding of ivabradine. Using homology modeling, we verified the presence of an inner cavity below the channel pore and identified residues lining the cavity; these residues were replaced with alanine (or valine) either alone or in combination, and WT and mutant channels were expressed in HEK293 cells. Comparison of the block efficiency of mutant *vs* WT channels, measured by patch-clamp, revealed that residues Y506, F509 and I510 are involved in ivabradine binding. For each mutant channel, docking simulations correctly explain the reduced block efficiency in terms of proportionally reduced affinity for ivabradine binding. In summary our study shows that ivabradine occupies a cavity below the channel pore, and identifies specific residues facing this cavity that interact and stabilize the ivabradine molecule. This study provides an interpretation of known properties of f/HCN4 channel block by ivabradine such as the “open channel block”, the current-dependence of block and the property of "trapping" of drug molecules in the closed configuration.

## Introduction

Cardiac f- and their neuronal relatives h-channels play a key role in the control of heart rate and neuronal excitability. These channels have a tetrameric composition, with single subunits belonging to the Hyperpolarization-activated Cyclic Nucleotide-gated (HCN) channel family. The HCN family includes 4 members (HCN1-4) that are differentially expressed in excitable tissues [1]. Each HCN subunit is organized according to a six transmembrane (S1–S6) structure, with an additional C-terminal cytosolic regulatory domain involved in cyclic nucleotide binding (CNBD) [1]. As in KcsA, Shaker, and HERG channels, also in HCN channels four subunits assemble to form a conduction pathway formed by the selectivity filter, a relatively large cavity lined by hydrophobic residues and the activation gate [2]–[6].

As well as playing a basic role in cardiac pacemaking, HCN channels have several important functions in neurons [7]. Increasing evidence suggests that the development of drugs targeting HCN channels may be useful as perspective bradycardic, anthiarrhythmic, anticonvulsant, analgesic and anaesthetic compounds [8]. Ivabradine is the first member of the “heart rate-reducing” family (I_f_ blocking agents) clinically approved by the European Medicines Evaluation Agency for the treatment of angina and heart failure. Given its clinical use, it is important to understand the molecular details of ivabradine block of HCN4 channels, the main isoform expressed in the pacemaker region of the heart. Some of the basic properties of the molecular interaction between ivabradine molecules and native f/HCN channels have been already clarified. For example it is known that drug molecules act intracellularly [9], and that the block is strongly state-dependent since it can only occur after channel opening [9]–[11]. In a study of mHCN1 block by ZD7288, another HCN blocking molecule interacting with pore-lining channel residues, Shin *et al*. [2] have shown that blocking molecules are "trapped" within channels in the closed state. Block of HCN4/f channels by ivabradine also has the unusual property of being "current-dependent", since it depends upon the flow of ions through the pore (kick-in/kick-off mechanisms). A tentative interpretation of this phenomenon predicts that the positively charged quaternary N^+^ ion of ivabradine antagonizes Na^+^/K^+^ permeating ions in their binding sites in the pore [9]–[11], but no evidence has been provided yet to support this hypothesis.

Despite the understanding of the basic features described above, no information is available yet on the specific interaction between ivabradine and the residues of the HCN4 channel and on the molecular details of block. We therefore set out to investigate the HCN4 channel block by ivabradine with three complementary approaches: 1) *in silico* analysis through homology molecular modeling (seeking information on the 3D structure of the channel pore); 2) mutagenesis and electrophysiological characterization of the interaction between ivabradine and mutated HCN4 channels; 3) *in silico* molecular docking, providing insight into the drug binding mode. Our data identify some specific residues in the S6 domain lining the internal mouth of channel pore acting in concert to bind ivabradine and provide a molecular-based explanation of known features of block.

## Materials and Methods

### Homology Modeling

**Figure 1:**
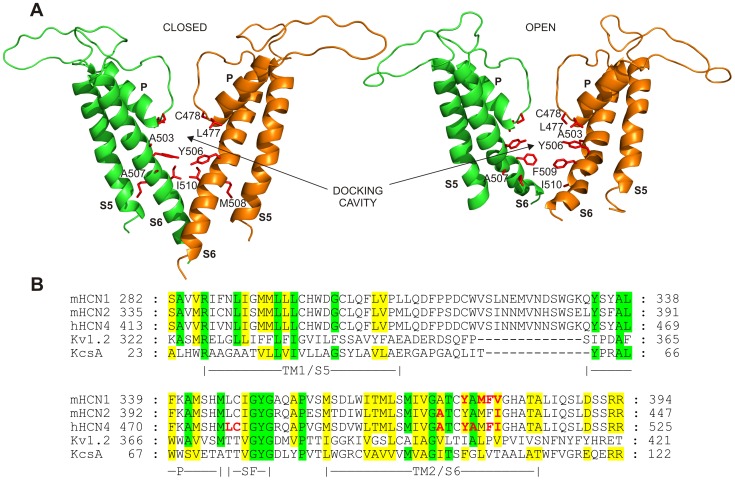
Homology models of the hHCN4 channel. A: Homology models of the TM1/S5-P-TM2/S6 regions of the hHCN4 channel in the closed (left) and open form (right), respectively. Only two of the four subunits are shown for clarity. Residues lining the internal cavity are shown in stick mode and labeled. M508 is also indicated even if it does not point towards the cavity. B: sequence alignment of the TM1/S5-P-TM2/S6 regions of hHCN4 with the corresponding regions of mHCN1, mHCN2, *Streptomyces lividans* KcsA, and the mammalian K^+^ channel Kv1.2. The secondary structure elements, as defined in the crystal structure of KcsA, are indicated. P is pore, SF is selectivity filter. Residues of HCN channels identical and similar to those of KcsA and/or Kv1.2 are highlighted by green and yellow boxes, respectively. Residues that face the internal cavity of the hHCN4 channel in the open and closed forms and residues relevant to mHCN2 block by cilobradine [24] and to mHCN1/mHCN2 block by ZD7288 [2],[24],[25] are indicated in red bold font.

In the absence of the crystal structure of the hHCN4 channel, a homology model of the pore region (S5-P-S6 region) of hHCN4 was obtained based on the *Streptomyces lividans* K^+^ channel (KcsA) X-ray structure, both in the closed (PDB-code 1BL8; [12]) and open forms (PDB-code 3FB7; [13]) (Fig. 1A,B). Sequence alignment was performed by first identifying potential transmembrane regions of the HCN sequences spanning the S5-P-S6 region with the programs TopPread 2 (S5: V419-V439; S6: V492-H512) [14], TMHMM v2.0 (S5:V419-M441; S6: L494-L516) [15], and HMMTOP (S5: I418-L442; S6: V492-A515) [16]. These regions were then used to guide the sequence alignment with the TM1-P-TM2 region of KcsA performed by CLUSTALW [17]. While the alignment of the HCN S6/TM2 region was readily achieved (based on the register provided by the GYG motif in the selectivity filter SF), the alignment of the predicted HCN S5/TM1 helix was not trivial, primarily because of the long S5/TM1-P linker in HCN and limited amino acid conservation with KcsA. For this reason we introduced a third sequence, from the rat Kv1.2 channel, to provide additional information useful to search for the proper register among KcsA, HCN4 and Kv1.2 S5/TM1. The KcsA and Kv1.2 sequences were first structurally aligned by superimposing their structures in the open state (PDB-codes 3FB7 and 2A79, respectively). The boundaries of S5/TM1 helix where then defined by identifying the conservation of HCN4 A414, R417 (in the first turn of the S5/TM1 helix in both KcsA and Kv1.2), L421, L426, and G433, thus positioning HCN4 P440 at the C-terminus of the S5/TM1 helix, as expected for a proline residue. A long gap was inserted between the S5/TM1 and the pore-helix in the KcsA sequence to align the predicted S5/TM1 and S6/TM2 helices of the HCN channels with those of the template (Fig. 1B). This alignment maximizes the overlap between TM prediction and sequence similarity to KcsA and Kv1.2 channels.

The tetrameric channel template was reconstructed by applying the appropriate crystallographic symmetry operations to the crystal structures 1BL8 and 3FB7. Then, the modelling of the tetrameric hHCN4 channel was performed by the program Modeller9v3 [18] using the model-multichain symmetry option. Ten models were generated and evaluated by using the discrete optimized protein energy (DOPE) score [19]. The best model was energy-minimized using the optimize procedure as implemented in the Modeller9v3 program and the stereochemistry further optimized by the stereochemical idealization procedure as implemented in the program REFMAC [20]. The program Procheck [21] was used to assess stereochemical quality.

Similar protein modeling was adopted for the hHCN4 mutants Y506A, I510A, and Y506A-I510A, with the channel in the closed form, and for the F509A mutant with the channel both in the open and closed form.

### hHCN4 Mutagenesis and Expression in HEK293 Cells

Point mutations were introduced into hHCN4 cDNA using QuikChange® II XL site-directed mutagenesis kit (Stratagene) and confirmed by DNA sequencing.

Human Embryonic Kidney cells (HEK 293, Phoenix) were cultured in Dulbecco’s MEM plus GlutaMAX™-I supplemented with 10% fetal bovine serum (GIBCO BRL) and antibiotics (PenStrept, SIGMA, Italy) at 37°C in 5% CO_2_. Expression vectors (pCDNA1.1) containing either the wild type (WT) or mutated hHCN4 cDNAs and a vector with the green fluorescent protein (pmaxGFP, Amaxa Biosystems) were co-transfected in HEK cells using either the Lipofectamine™ Reagent (Invitrogen) or the *FuGENE*® *HD* Transfection Reagent (Roche). Cells were incubated at 37°C (5% CO_2_) for 2–3 days to allow for a good level of protein expression prior to electrophysiology experiments. On the day of the experiment, cells were detached and dispersed by trypsin and plated at a low density on a 35 mm plastic Petri dish. The dish was then placed under the stage of an inverted fluorescence microscope, and GFP-expressing cells were selected for voltage-clamp analysis.

### Voltage-clamp Recordings

All experiments were carried out in the whole-cell configuration at the temperature of 32±0.5°C. The recording pipettes contained (in mM): NaCl 10, K-Aspartate 130, MgCl_2_ 0.5, EGTA-KOH 1, HEPES-KOH 1, ATP (Na-salt) 2, GTP (Na-salt) 0.1, phosphocreatine 5, (pH 7.2). The control extracellular solution contained (in mM): NaCl 140, KCl 5.4, CaCl_2_ 1.8, MgCl_2_ 1, D-glucose 5.5, HEPES-NaOH 5, (pH 7.4); BaCl_2_ 1 mM, and MnCl_2_ 2 mM were added to improve dissection of the HCN current. Ivabradine was added to the extracellular solution by dissolving a stock solution (10–50 mM) to the desired final concentration. Currents were recorded and filtered online at a corner frequency of 1 KHz with an Axopatch 200B amplifier, and acquired using the pClamp 10.1 software. Activation curves for HCN currents were obtained by the following voltage protocol: from a holding potential of −25/−35 mV test voltage steps ranging from -40 to -145 mV (15 mV interval) were applied until steady-state current activation was attained at each potential; test steps were then followed by a pulse to −130 (or −145 mV in some protocols) and by a deactivating pulse to +10 mV. Time constants of activation (at −140 mV) and deactivation (at +5 mV) were obtained by fitting with a monoexponential function the time-dependent current traces; the initial delay [22] was ignored.

Ivabradine block of WT hHCN4 channels was investigated by superfusing the drug during repetitive (0.5 Hz) application of activating (−140 mV, 0.6 s)/deactivating (+5 mV, 0.3 s) steps, from a holding potential of −35 mV. The fractional current block was calculated as the ratio between block-induced current reduction and control current (at −140 mV). For some mutant currents this protocol was modified to account for changes in the current kinetics as follows: for mutants Y506A, A507V, F509A, Y506A-F509A the duration of the activating step was increased to 1 s; for mutant I510A the duration of the activating step was shortened to 0.2 s; for mutant F509A the deactivation step duration and the frequency of stimulation were set to 2.5 s and 0.25 Hz, respectively, to ensure complete deactivation of the channel. Dose-response relationships were obtained by fitting experimental data with a Hill equation (Y = Y_max_*(1/(1+ (IC50/*x*)n_H_))). Only in those experiments where drug recovery was complete, a second dose of the drug was tested; in all other cases each cell was exposed to a single drug concentration.

All data are presented as mean±SEM values. Statistical analysis was performed with the Student’s t-test for unpaired data. Dose-response curves were compared using the Extra sum-of squares F test (GraphPad Prism 5). Significance was set at P<0.05.

### Ivabradine Docking in hHCN4 Wild-type and Mutant Channels

Docking of ivabradine to hHCN4 tetramer, both in the closed and open form, was performed with the program AutoDock4.0 [23] which combines a rapid energy evaluation through precalculated grids of affinity potentials with a variety of search algorithms to find suitable binding positions for a ligand on a given protein. When docking, the structure of hHCN4 was kept rigid, but all the torsional bonds in the ivabradine molecule were set free to perform flexible docking. Polar hydrogens were added by using the Hydrogens module in AutoDock Tools (ADT) for hHCN4; after that, Kollman united atom partial charges were assigned. Docking was carried out using the Lamarckian genetic algorithm, applying a default protocol for 100 independent docking runs. Results were clustered according to the 2.0 Å root-mean-square deviation (RMSD) criterion. The grid maps representing the proteins in the actual docking process were calculated with AutoGrid, with a spacing of 0.375 Å between the grid points. The grid size was chosen (and centered) to be sufficiently large to include the internal channel at the tetrameric hHCN4 interface. Similar ligand docking procedures were adopted for the hHCN4 mutants Y506A, I510A, and Y506A-I510A, with the channel in the closed form, and for the F509A mutant with the channel both in the open and closed form.

## Results

### Residues Facing the Water-filled Cavity

Previous results have suggested that ivabradine exerts its blocking action on f/HCN channels by binding to a site within the aqueous cavity in the inner mouth of the pore [10], [11]. A similar binding location has been proposed for mHCN2 block by cilobradine [24], a structural analog of ivabradine, and also for mHCN1/mHCN2 block by ZD7288, a structurally different molecule [2], [24], [25].

To identify residues potentially involved in drug binding, we first explored the spatial orientation of residues facing this inner cavity by means of an *in silico* homology 3D model of the pore region (S5–S6 region) of hHCN4. Homology models were obtained based on the *Streptomyces lividans* K^+^ channel (KcsA) X-ray structures, both in the closed [12] and in the open conformation [13] (Fig. 1A). We selected KcsA as a model rather than Kv1.2 (both are comparably similar to HCN4 in terms of sequence alignment) for three main reasons: (i) the KcsA structure is known in both the closed and open forms, while only the open structure is available for Kv1.2; (ii) the KcsA structure has been already used successfully as a model template for the HCN2 channel [24]; (iii) the C-terminal part of the TM2/S6 helix in Kv1.2 hosts two almost consecutive proline residues (P405, P407) which confer to the helix a structure divergent from that of KcsA, where these proline residues are missing (Fig. 1B). Since HCN sequences do not have proline residues in the TM2/S6 helix, and prolines usually alter the normal H-bonding pattern of helices, the choice of KcsA as a model for HCN4 seemed more appropriate.

Both the KcsA-based open and closed models delineate a cavity lined by residues of the S6 segments and by the lower part of the pore (P) region, which includes the selectivity filter (SF). More specifically, in the closed configuration model the inner cavity of the hHCN4 channel pore lays below the selectivity filter and is lined by residues L477, C478, A503, Y506, A507, and I510. Note that the side chains of residues Y506 and I510 are arranged in a sort of double-layer crown which delimits the floor of this cavity (Fig. 1A, left). In the open form, the inner cavity is lined by residues L477, C478, A503, Y506, A507, F509, and I510, and the diameter of the channel at the cytosolic entrance is ∼6.5 Å larger than in the closed structure (distance calculated between I510 side chains of opposite subunits). In the open conformation the floor of the inner cavity, even if looser, is still delimited by a double-layer crown of residues (Y506 and F509, Fig. 1A, right). In Fig. 1B the sequence alignment of the S5-P-S6 regions of hHCN4 with the corresponding regions of mHCN1, mHCN2, KcsA, and Kv1.2 is shown. The alignment reveals that most of the hHCN4 residues facing the internal cavity (L477, C478, A503, Y506, A507, F509, and I510, bold red) match those previously reported to be involved in ZD7288 and cilobradine interactions with mHCN2 (A425, I432, bold red) [24] and mHCN1 (C347, Y375, M377, F378, V379, bold red) [2], [25]. It is important to note that while residue M377 of mHCN1 is reported to be potentially involved in the blocking action of ZD7288 [2], the corresponding residue (M508) in our models points towards the S5–S6 interface and does not line the internal cavity in either closed or open forms of the hHCN4 channel (Fig. 1A). Based on the above modeling information, we generated single mutants by replacing L477, C478, A503, Y506, A507, M508, F509, and I510 with alanine or, when the native residue was an alanine, with valine, in order to investigate which of the potentially interfering residues are involved in ivabradine binding.

### Ivabradine Action on hHCN4 WT and Mutant Channels

All mutations, except L477A were associated with functional channels when expressed in HEK293 cells and the biophysical properties are listed in Table S1; as shown in Fig. S1 and Table S1, the L477A mutant was normally expressed in the plasma membrane, but did not generate functional currents.

As apparent from the data in Table S1, some of the mutants underwent significant changes in the voltage dependence of gating (V_1/2_ and time constants of activation/deactivation). Since the blocking action of ivabradine (30 µM) on hHCN4 WT and mutant currents was evaluated during trains of activating/deactivating steps (−140/+5 mV), voltages at which all types of channels were fully open or fully closed, respectively, these changes did not affect the measurement of mean steady-state ivabradine block.

**Figure 2:**
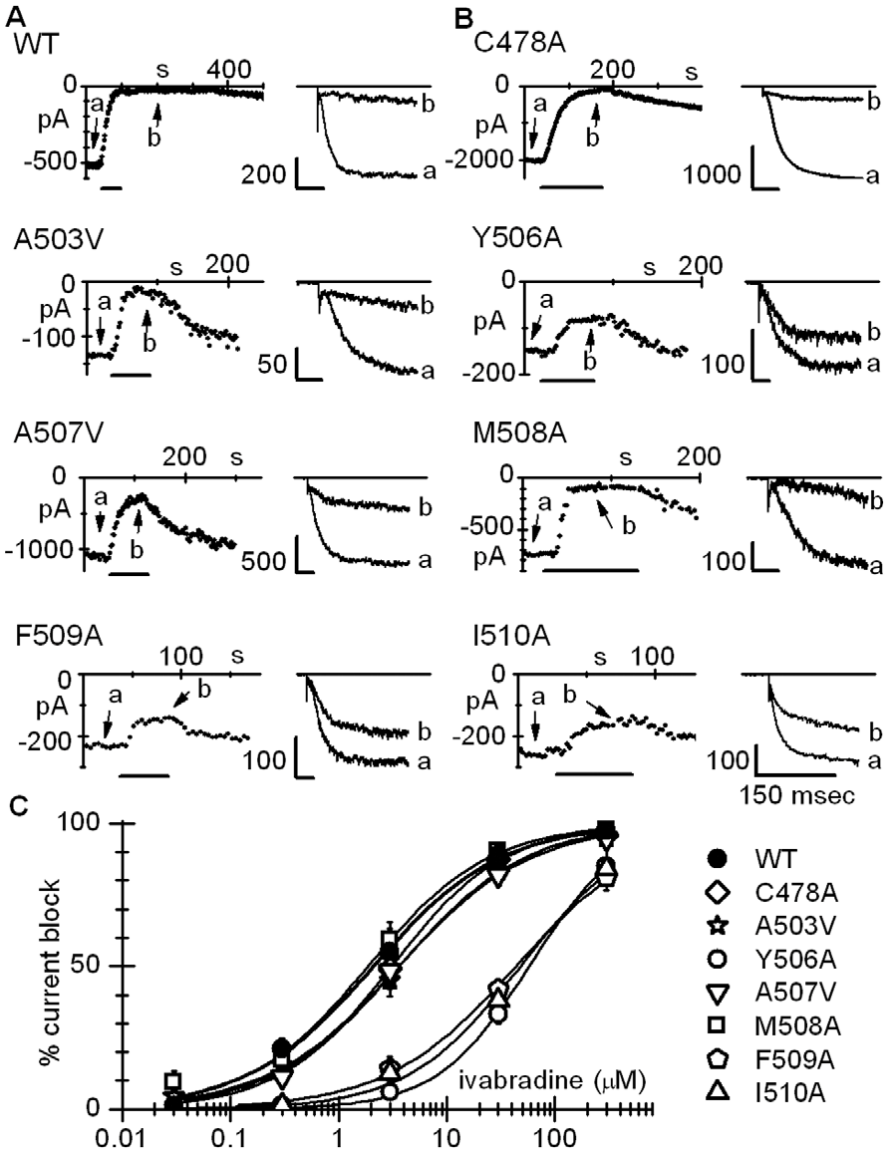
Ivabradine-induced block of WT and mutant hHCN4 channels. A,B: time-courses of current amplitudes (left panels) and sample current traces (right panels: a, control; b, steady-state block) recorded at −140 mV before, during, and after superfusion with 30 µM ivabradine (horizontal bars). Horizontal bars in right panels: 150 ms. C: Dose-response relations of ivabradine block of WT and mutant channels. Fitting with the Hill equation (curves) yielded half-block concentrations (IC_50_) of 2.1, 3.1, 3.6, 57.7, 3.7, 1.9, 44.0, 47.7 µM and Hill coefficients (n_H_) of 0.74, 0.84, 0.72, 1.02, 0.75, 0.77, 0.73, 0.83 for WT, C478A, A503V, Y506A, A507V, M508A, F509A, and I510A channels, respectively. Dose-response relations for Y506A, F509A, and I510A curves are significantly different from WT (P<0.05).

The time-courses of current amplitude at −140 mV during drug superfusion and sample current traces in control (a) and at steady-state block (b) are shown in Fig. 2A,B, left and right panels, respectively. In all cases ivabradine caused a reduction of the current which accumulated over time until steady-state block was attained, normally within the first 50–100 s of drug perfusion. Mean steady-state percent block values were: 88.8±1.6% (n = 7), 87.6±3.4% (n = 5), 83.6±1.9% (n = 5), 33.3±3.5% (n = 5), 81.9±3.8 (n = 5), 90.3±2.0% (n = 6), 41.9±2.1% (n = 5), and 38.0±3.1% (n = 4) for WT, C478A, A503V, Y506A, A507V, M508A, F509A, and I510A channels, respectively. Statistical analysis revealed that ivabradine blocks C478A, A503V, A507V, and M508A channels as efficiently as WT hHCN4 channels, but exerts a significantly less efficient block on Y506A, F509A, and I510A channels (t-test, P<0.05 *vs* WT channels). For fuller comparison, we extended the investigation of ivabradine-induced block to a wider range of drug concentrations. The resulting dose-response curves and fitting parameters are shown in Fig. 2C. The mutant dose-response curves clearly fall into two categories, those which essentially overlap the hHCN4 WT curve (C478A, A503V, A507V, and M508A) and those which show a much reduced (some 20/30-fold lower) sensitivity to the drug (Y506A, F509A, and I510A). These data indicate that while C478A, A503V, A507V, and M508A mutations do not affect the efficiency of hHCN4 channel block by ivabradine, reduced block efficiency is obtained with the mutations Y506A, F509A, and I510A. This suggests that residues Y506, F509, and I510 may be involved in the drug-channel interaction in agreement with the homology modeling data indicating that the same residues face the internal surface of hHCN4 pore and form the double layer crown composing the floor of the cavity in the closed (Y506 and I510) and open (Y506 and F509) states (Fig. 1A).

**Figure 3:**
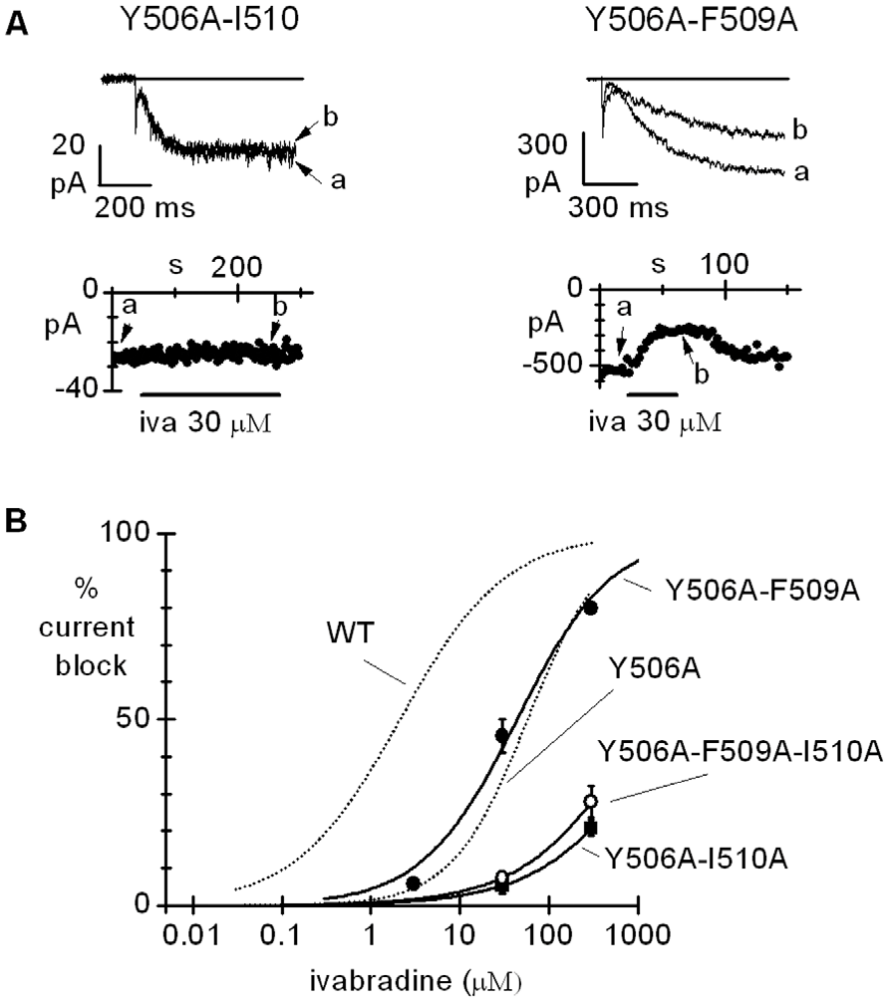
Block of double (Y506A-I510A and Y506A-F509A) and triple (Y506A-F509A-I510A) mutant channels by ivabradine. A, top, sample currents recorded at −140 mV from the two double mutant channels Y506A-I510A and Y506A-F509A in control (a) and after steady-state block by ivabradine 30 µM (b); A, bottom, time course of currents at −140 mV during ivabradine perfusion. B: dose-response relations; Hill fitting (full lines) yielded IC_50_, nH values of 2213.0 µM, 0.7, 42.5 µM, 0.8, and 1215.0 µM and 0.68 for Y506A-I510A, Y506A-F509A, and Y506A-F509A-I510A respectively. Hill fitting of WT and Y506A block from Fig. 2 also drawn for comparison (dotted lines). The two double-mutant curves are significantly different between themselves and from that of WT channels (P<0.05); the Y506A-I510A and the Y506A-F509A-I510A curves are not significantly different (P<0.05).

Based on these observations, we proceeded to verify the efficacy of ivabradine block of the double mutants Y506A-I510A and Y506A-F509A. Both double mutants elicited functional currents. Properties of the double-mutant currents are provided in Table S1. The time-courses of current amplitude at −140 mV during perfusion with ivabradine 30 µM and sample current traces in control (a) and at steady-state block (b) are shown in Fig. 3A, as indicated, for the double-mutant channels investigated. The dose-response curves of ivabradine block in Fig. 3B show that while the Y506A and I510A mutations have a cumulative effect (IC_50_ = 2213.0 µM) this does not occur for the Y506 and F509A mutations (IC_50_ = 43.7 µM). This seems to suggest that a structural re-arrangement essential to determine ivabradine sensitivity is common to Y506A and F509A, and that once this has been rendered by Y506A, further mutating F509 does not provide a supplemental effect.

We also tested the effect of the triple mutation Y506A-F509A-I510A and verified that the blocking affinity of ivabradine for this channel is similar to that of the double mutant Y506A-I510A (IC50 of 1215.0 and 2213.0 µM, respectively; P>0.05, Fig. 3B); this further supports the lack of cumulative action of F509A. Thus, mutation F509A is effective when alone, but not when in combination with Y506A or Y506A-I510A.

**Figure 4:**
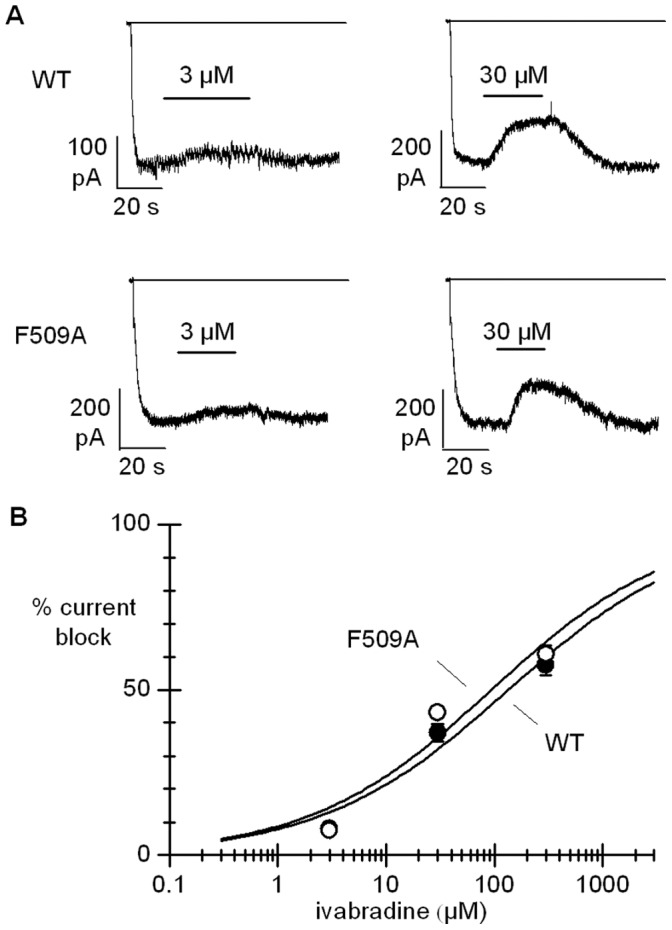
Ivabradine block of WT and F509A channels in the open state. A, representative traces showing the action of ivabradine (3 and 30 µM) on the current recorded from WT and F509A channels during a long (100 s) step to −100 mV. B, dose-response relations for ivabradine block measured as in A. Hill fitting resulted in IC_50_, nH values of 120.7 µM, 0.5 and 91.3 µM, 0.5 for WT (filled circles) and F509A channels (open circles), respectively (non-significantly different, P>0.05).

As shown in Fig. 1, F509 points toward the inner cavity only in the open channel. To gain more insight into the role of F509 we used a protocol allowing evaluation of ivabradine-induced block of the F509A mutant specifically in the open channel configuration. In Fig. 4A sample current traces recorded at steady-state activation during long (∼100 s) hyperpolarizing steps to −100 mV in the presence of 3 and 30 µM ivabradine for WT (top) and F509A (bottom) channels are shown. The full dose-response curves are shown in Fig. 4B; Hill fitting of experimental data yielded IC_50_ of 120.7 µM and 91.3 µM for the wild type and F509A mutants, respectively (not significantly different, P>0.05).

The results in Fig. 4 imply that the F509A mutation does not modify block developing when channels are in the open state, and agree with the hypothesis apparent from the data in Fig. 3 that the F509A mutation affects the closed channel state, possibly through the same structural rearrangement associated with the mutation Y506A.

### Ivabradine Docking in WT hHCN4 Channels

The results in Figures 2, 3, and 4 indicate that residues Y506, F509, and I510 are important determinants of hHCN4 channel block by ivabradine, with a cumulative action observed for residues Y506 and I510, but not for residues Y506 and F509, and a higher efficiency of block predicted for the closed conformation of channels. The question whether or not these residues directly interact with ivabradine was addressed by means of an *in silico* docking approach using a homology model structure of the hHCN4 tetrameric pore region. A similar approach was recently applied to describe the block of HCN2 channels by the drug ZD7288 [24].

**Figure 5:**
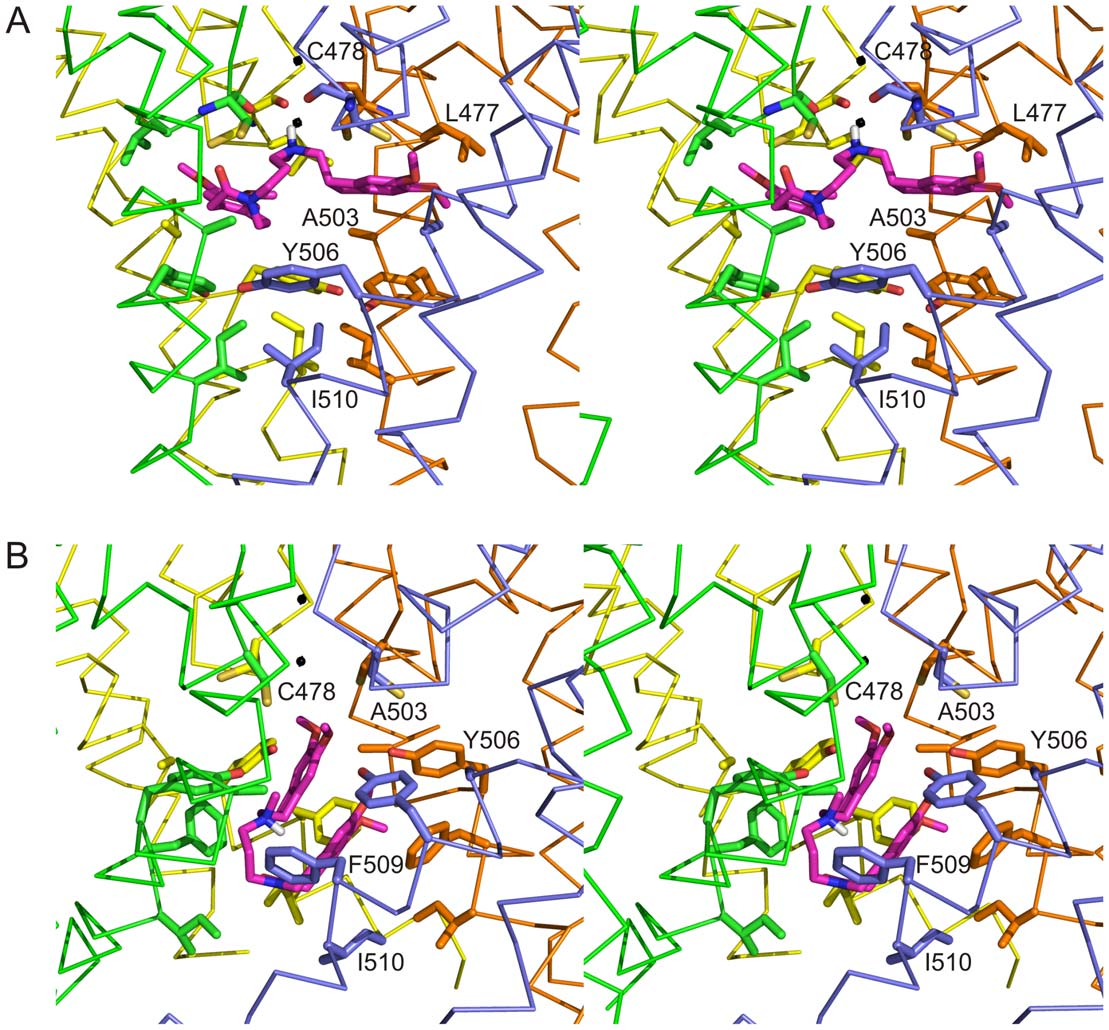
Detailed stereo view of the ivabradine docking in the hHCN4 WT channel. Stereo view of the interior of the WT hHCN4 channel in its closed (A) and open (B) form with the best-docked pose of ivabradine shown in magenta. The S5-P-S6 regions of the four hHCN4 subunits are shown as ribbon in green yellow, orange and pale blue, respectively. Side-chains of residues interacting with the ivabradine molecule are shown in stick representation in both panels. For the closed state only, the main-chain atoms of C478 are shown since the carbonyl oxygen atoms may form additional H-bonds with ivabradine. I510 is also shown in the channel closed form, though this residue does not interact directly with ivabradine. Black spheres indicate positions corresponding to K^+^ ion bound in pore of the KcsA crystal structure [12]. For clarity only residues of one subunit are labeled and L477 of the pale blue subunit is omitted.

We first analyzed the docking of ivabradine to the hHCN4 WT channel in its closed state using the estimated Free Energy of Binding (ΔG_b_) as the scoring function. The best clustered docked models (consisting of 4 models over 100 trials) display an average ΔG_b_ of −10.2 kcal/mol, with the bound ivabradine having its quaternary N atom approximately along the axis of the channel pore and the benzazepinone and benzocyclobutane moieties localized in two of the four hydrophobic pockets lined by L477, C478, A503, and Y506 from different subunits (Fig. 5A). The docked ivabra\dine molecule is stabilized by several van der Waals and hydrophobic interactions with at least one of its heterocyclic moieties (both in the best ivabradine docking pose) making stacking interactions with the Y506 side-chains which build the floor of the cavity (Fig. 5A); only one ivabradine molecule is hosted in the cavity. Note that the average distance of the ivabradine quaternary N atom from the innermost permeating ion binding position (lowest dot in Fig. 5A) is 2.6 Å, calculated over the 4 models of the best docking cluster; this distance reduces to only 1.5 Å for the best-docked pose (ΔG_b_ = −10.5 kcal/mol) within the best docking cluster (Fig. 5A). In this latter case the positively charged ivabradine quaternary N atom lies at a distance of 3.1–3.7 Å from each of the four carbonyl oxygen atoms of C478 of the selectivity filter, thus suggesting that the best stabilization of the bound ivabradine to the tetrameric closed channel may include additional H-bonds during the dynamic ligand-receptor interactions.

An important observation emerging from the docking analysis is the structural role played by residues Y506 and I510. In the tetrameric hHCN4 closed channel model, the four Y506 side-chains point to the interior of the channel and form the floor of the cavity hosting ivabradine (Figs. 1A left, 5A). Furthermore, I510 residues fall right below Y506 residues, and by means of hydrophobic interactions may stabilize the orientation of the Y506 side-chains (Figs. 1A left, 5A).

We then analyzed ivabradine docking to the hHCN4 WT channel in the open form. As pointed out in Fig. 1A, right, when the channel is open, the internal cavity takes a more relaxed and enlarged conformation. The best clustered ivabradine docked models (2 models over 100 trials) showed an average ΔG_b_ = −8.04 kcal/mol, a value smaller than that estimated for the closed WT hHCN4 channel (ΔG_b_ = −10.2 kcal/mol). In the open channel, docked ivabradine molecules adopt a bent conformation, with the benzazepinone and benzocyclobutane moieties almost parallel to each other and stabilized by stacking interactions to the aromatic side-chains of Y506 and F509 (Fig. 5B). The ivabradine quaternary N atom is now positioned far below the permeating ion binding site (distance of 8.5 Å calculated over the 2 docking solutions of the best cluster), in the region of the channel lined by the F509 side-chains.

### Ivabradine Docking in hHCN4 Mutant Channels

**Figure 6:**
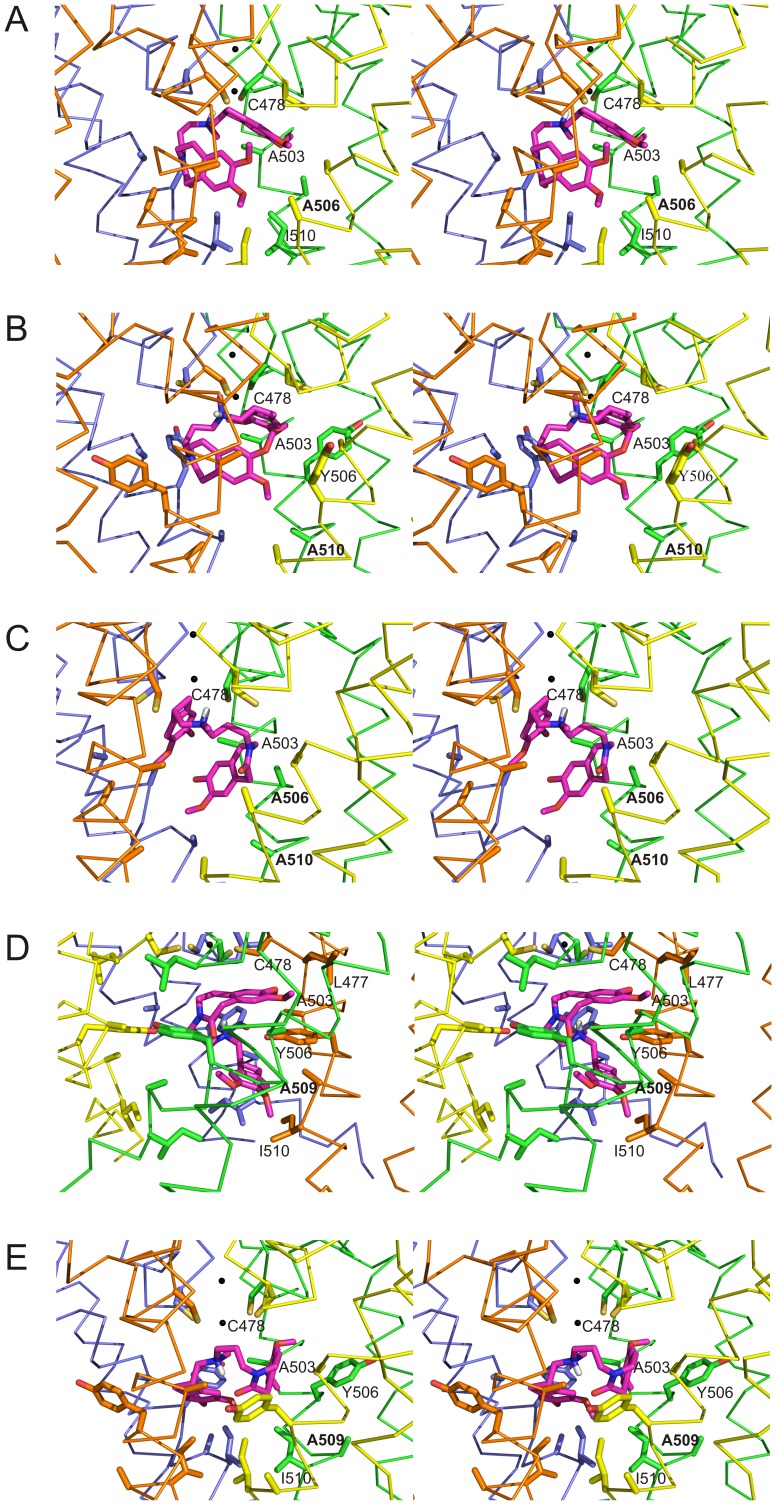
Detailed stereo view of the ivabradine docking to hHCN4 mutant channels. A,B,C: stereo views of the interior of hHCN4 mutants Y506A (A), I510A (B), and Y506A-I510A (C) in the closed form. D, E: stereo views of the interior of the F509A mutant in the open (D) and closed (E) form. Side-chains of residues relevant to ivabradine binding are shown as ball-and-stick in all panels. In all mutant models the best pose of the docked ivabradine is shown in magenta, while the S5-P-S6 regions of the four hHCN4 subunits are shown as ribbon in grey, yellow, orange and pale blue, respectively. For clarity, residues have been labelled only in one hHCN4 subunit, with mutated residues indicated in bold characters.

To verify if modeling is able to explain changes in block efficiency as those found in electrophysiology experiments with mutant Y506A and I510A channels (Figures 2 and 3), we performed additional modeling and docking calculations for the two single mutants Y506A and I510A and for the double mutant Y506A-I510A (Fig. 6). Inspection of the Y506A mutant model structure indicates that mutation increases the volume of the docking cavity (Fig. 6A); the floor of the ivabradine docking cavity is now built by the I510 side-chains. The best clustered docking models for Y506A mutant indicate for ivabradine a bent conformation with the benzazepinone and benzocyclobutane moieties almost orthogonal to each other, with one moiety located in the pocket lined by C478, A503, and the mutated A506, and the other moiety fitting most of the volume which in the hHCN4 WT channel is occupied by the Y506 side-chains, close to I510 side-chains. The best docking cluster (3 models over 100 trials) for the Y506A mutant, with the channel in the closed form, show an average ΔG_b_ = −8.02 kcal/mol, a value smaller than that estimated for the closed WT hHCN4 channel (ΔG_b_ = −10.2 kcal/mol). This indicates that the lack of the Y506 side-chain decreases the stabilization of the bound ligand and allows for a different spatial position of ivabradine, with its quaternary N atom displaced from the center of the pore and more distant from the lowermost permeating ion binding site (4.3 Å averaged over the 3 models of the best solution cluster).

When we modelled the I510A mutant, we noticed that the loss of the hydrophobic interactions between Y506 and I510 side-chains allows the Y506 side-chain of each subunit of the tetrameric channel to re-orient away from the centre of the pore (Fig. 6B). Indeed, the best I510A mutant model shows that now each Y506 side-chain is located in a subunit-subunit interface, in a cavity lined by L447, G502, and F509 from one subunit, and I510, A503, and T504 from the adjacent subunit. Such new orientation of Y506 determines a structure of the lower part of the channel and a docking behaviour of ivabradine similar to that found in the Y506A mutant (Fig. 6A, B), with the best docking cluster (3 models over 100 trials) having an average ΔG_b_ = −8.29 kcal/mol. This agrees with the evidence above that both the Y506A and I510A single mutants are characterized by a much lower affinity to ivabradine than both the WT and any other single mutant hHCN4 channel (Fig. 2).

Modelling of the Y506A-I510A double mutant shows that the double crown side-chain layer making the floor of the internal cavity in WT hHCN4 closed channel is now completely removed, which allows for an enlarged volume of the docking cavity at the cytosolic side of the channel (Fig. 6C). As a consequence, the docked ivabradine molecule can span a wider number of conformations, with the benzazepinone and benzocyclobutane moieties now fitting in the cavity lined by C478, A503, and A506 side-chains and in the cavity below lined by A503, A506, and A510, respectively. The best docking cluster for the double mutant (3 models over 100 trials) has an average ΔG_b_ = −6.7 kcal/mol, much smaller than those of the hHCN4 WT and of Y506A and I510A single mutants, with the best docking-pose of ivabradine in a bent conformation reminiscent of that found in the Y506A and I510A mutants (Figs. 6A,B,C).

We then further analyzed the role of the F509 residue by means of modeling and docking experiments with the F509A mutant in both the open and closed forms. The open form of the F509A mutant channel shows a more enlarged conformation of the lower part of the channel cavity when compared to that of the WT hHCN4 (Fig. 6D). The best docking cluster (3 models over 100 trials) shows an average ΔG_b_ = −7.16 kcal/mol. In the F509A open channel mutant the docked ivabradine molecule adopts an elongated conformation along the channel axis with the benzazepinone and benzocyclobutane moieties almost orthogonal to each other, the first hosted in the pocket lined by L477, C478, A503, and Y506 and the second in the pocked lined by Y506, A509 and I510 (Fig. 6D). ΔG_b_ values calculated for the open WT and F509A channels are comparable (−8.04 *vs* −7.16 kcal/mol), which agrees with the electrophysiological evidence that the residue F509 is not relevant to the open channel block (Fig. 4). Interestingly, when we modelled the F509A mutant in the hHCN4 closed form, we observed that the absence of the F509 side-chain promotes an overall rearrangement of the surrounding structure. Indeed, as shown in Fig. 6E and Fig. S2, the removal of the F509 side-chain makes room for the Y506 side-chain of each subunit, which may thus move from the centre of the pore towards the subunit-subunit interface as seen in the I510A mutant (Figs. 6B). For the best ivabradine cluster results (6 models over 100 trials) the average ΔG_b_ = −8.95 kcal/mol is similar to that found for the I510A mutant (ΔG_b_ = −8.29 kcal/mol) and smaller than that found for WT hHCN4 (ΔG_b_ = −10.2 kcal/mol).

Modeling thus provides an explanation for the block data obtained with the F509A mutation (Figs. 1 to 4) and confirms the hypothesis that the reduction of block sensitivity for this mutant is an indirect effect associated with the displacement of residue Y506.

## Discussion

The relevance of the I_f_ current to cardiac pacemaker generation and modulation of rate has been documented both in experimental animals and in humans where it represents an important pharmacological target [26]. A detailed understanding of drug-channel interaction is therefore essential in the perspective of improving HCN isoform-specific selectivity of block, particularly since differential isoform distribution in heart and brain may underlie isoform-dependent pathologies including for example arrhythmias, epilepsy, motor learning defects, pain transmission [7], [8], [27], [28]. We therefore sought to investigate details of ivabradine-induced block by identifying residues involved in the binding of ivabradine to HCN4, the HCN isoform most highly expressed in the sinoatrial node. Previous studies have suggested that the cytoplasmic side of HCN channels is composed of a water-filled cavity guarded by an intracellular activation gate and that the binding site for HCN blocker drugs such as ZD7288, ivabradine, and cilobradine is located within this central cavity [2], [3], [10], [11], [24], [25]. Although these studies have indicated the involvement of some specific residues, they did not provide an integrated and detailed description of the structural arrangement of the cavities in the channel open and closed states, and how this arrangement affects ivabradine block.

The homology models shown in Fig. 1A aim to fill this gap by providing structural information on the spatial organization of residues in the S6 domain and in the S5–S6 linker that line the internal cavity in both open (L477, C478, A503, Y506, A507, F509, and I510) and closed (L477, C478, A503, Y506, A507, and I510) channel states. A similar 3D modeling approach, has been previously proposed for mHCN2 [24], though limited to the open state of the channel. According to Cheng *et al.*’s study [24], mHCN2 residues A425 and I432 (structurally homologus to A503 and I510 of hHCN4) face the internal cavity of channels, in agreement with our data.

As shown by our previous studies [10], [11], the mechanism of action of ivabradine block is quite complex. The drug can only access its binding site, located in the internal channel cavity, when f/HCN4 channels are in the open state. Also, block is current-dependent since the inward current flow at hyperpolarized potentials removes block (kick-off), while depolarization favors block development (kick-in); this mechanism is responsible for the use-dependence of ivabradine. In addition, the property of current-dependence suggests that the ionic flow through the channel pore proceeds according to a multi-ion single file permeation model, and that the charged nitrogen of ivabradine competes with permeating Na^+^/K^+^ ions at one of the coordination sites along the permeation pathway, most likely the innermost one [10]. Since the present interpretation of mechanisms underlying the interaction between ivabradine and HCN channels relies essentially only on electrophysiological data, we used a combination of alanine-scanning mutagenesis and 3D-modeling and docking in the attempt to resolve the molecular basis of channel block.

The first important information yielded by the modeling approach is the indication that when the channel is in the open state, the smallest diameter of the internal mouth of the cavity is of about 11 Å (calculated between F509 side-chains of opposite subunits), which is enough to allow ivabradine to access the cavity in a dynamic structural context. On the contrary, when the channel is in the closed state, ivabradine cannot enter since the smallest diameter of the internal cavity is about 4–5 Å (calculated either between the T514 or between the I510 side-chains of opposite subunits). These data thus provide an explanation for the known "open channel" block property of ivabradine [10] and for the "trapping" of blocking molecules within the channel in the closed state, a property described also for the block of mHCN1 by ZD7288 [2]–[6].

Electrophysiological analysis of mutant block by ivabradine indicate that the residues forming the floor of the closed cavity (Y506 and I510, Fig. 1A) are major determinants of ivabradine block measured during activation/deactivation protocols (Figures 2 and 3). These data indeed show that block is similarly reduced in Y506A and I510A mutant channels (Fig. 2; IC50 = 57.7 and 47.7 µM, respectively), and is further strongly reduced in the double mutant Y506A-I510A (Fig. 3, IC50 = 2213.0 µM). The structural position occupied by residues Y506 and I510 in hHCN4 appears to be critical for drug binding also in hERG channels, since the corresponding aminoacids (Y652 and F656) have been identified as critical elements for the interaction with several structurally unrelated drugs [29]–[31]. A molecular rationale to interpret the block data of HCN4 channels can be obtained from 3D-modeling. As shown in Fig. 5, the “best” pose of drug molecules within the channel differs substantially in closed and open channels. In the closed state, the position occupied by ivabradine is proximal to the internal pore end and is stabilized by a floor formed by the side-chains of residues Y506 and supported by I510 residues, representing the inner boundary of the channel cavity. In the open channel state the floor of the cavity is partly disassembled, and as a consequence the cavity is wider and ivabradine finds a stable binding site in a position farther away from the pore; given the larger opening of the inner cavity (about 11 Å), ivabradine molecules are not trapped anymore and move easily across it.

As discussed above, our simulations highlight a significant re-shaping of the docking cavity when channels change from the open to the closed state. As well as acquiring a more pore-proximal position relative to the open state, in the closed state drug molecules modify their orientation such that the quaternary N atom moves from a more peripheral to a more central position relative to the pore axis. In closed channels, the position acquired by the N-atom is sufficiently close to the lowermost of the binding sites of permeating ions (1.5 Å) to antagonize their binding. This provides a satisfactory explanation of the property of “current dependence” of block previously described [10]. 3D-docking model analysis indicates that the values of the ΔG_b_ of ivabradine binding to the two mutant Y506A and I510A closed channels are similar (−8.02 kcal/mol and −8.29 kcal/mol, respectively) and are both smaller than that for the WT closed channels (−10.2 kcal/mol). This agrees with the experimental evidence presented in Fig. 2 that block is similarly reduced for Y506A and I510A relative to WT, and further stresses the evidence that modeling and electrophysiological data converge in the interpretation of block features.

Docking analysis of ivabradine in WT closed channel (Fig. 5A) shows that the drug interacts directly with Y506 but not with I510. However I510 residues lie beneath the Y506 side chains and their interactions maintain the orientation of both residues toward the centre of the cavity. In the I510A mutant channel, these interactions are lost and, as a consequence, the Y506 side-chains can now change their orientation in a way which decreases the stabilization of the bound ligand (Fig. 6B).

Results obtained with the double mutant Y506A-I510A, where according to 3D modeling the integrity of the floor of the cavity is disrupted in the closed configuration (Fig. 1), concur to indicate that residues Y506 and I510 are major determinants of block efficiency (Fig. 3, 6C). Consistent with block data, the ΔG_b_ is smaller for the double mutant (−6.70 kcal/mol) than for either of the two single mutant channels.

As for the open channel, the experiments with double and triple mutants (Fig. 3) reveal that the role of the floor of the cavity, formed mainly by residues Y506A and F509A, is less critical. While block of the single F509A mutant is reduced, indicating a role for this residue in block determination, data in Fig. 3 and Fig. 4 rule out a direct contribution. According to the analysis of ivabradine docking to F509A channels (Fig. 6D, E), this can be explained by assuming that the F509 side-chain exerts a spatial constraint on the orientation of Y506, and its removal causes a rotation of the Y506 side-chain, an effect able to destabilize ivabradine binding. This is indeed verified when comparing the orientation of Y506 side-chains in the F509A mutant channel (Fig. S2).

### Comparison with Previous Studies

Our hHCN4 model can be compared with previous studies of other HCN channels based on homology modeling, scanning accessibility and/or drug interaction experiments.

Investigation of spHCN and mHCN1 channel block by ZD7288 has shown that residues homologous to hHCN4 Y506, M508 and I510 are accessible to drug block and therefore face the internal cavity of channels [2]. These results agree with our finding that Y506 and I510 face the pore, while in our model M508 does not line the internal cavity but rather points towards the S5–S6 interface. It is interesting to note, however, that Chan *et al.* [25] recently found that mHCN1 M377, homologous of hHCN4 M508, does not face the cytoplasmic side of the pore. The same authors [25] also reported that mHCN1 residues homologous to hHCN4 C478, C505, F509 and I510 face the internal cavity in agreement with our data.

mHCN2 residues homologous to hHCN4 A503 and I510 also face the internal cavity as in our model according to Cheng *et al.* [24]. Our hHCN4 model is also consistent with cysteine-scanning mutagenesis experiments showing that spHCN C428 (homologous to hHCN4 C478) faces the cytoplasmic side of the channel pore, while spHCN K433 (homologous to hHCN4 R483) faces the extracellular vestibule [32].

Further investigation by cysteine-scanning mutagenesis [3], [33] has shown that spHCN residue Q468, corresponding to hHCN4 Q518, is accessible in the closed channel state, while T464, corresponding to T514, is not. These results suggest that the narrowest region of the crossing bundle lies in the vicinity of the end of S6. Giorgetti *et al*. [4] identify spHCN Q468 as the position of narrowest opening of the spHCN crossing bundle in the closed channel state (their Fig. 8). Although we have not performed accessibility experiments, our homology 3D model predicts that the narrowest region of the channel (in the closed state) is located close to T514 (thus slightly upstream of that reported for spHCN channels) with Q518 protecting T514 from the solvent at the cytosolic side. In our model Q518 is therefore accessible even in the closed channel state, while T514 is not.

Giorgetti *et al*. [4] used the crystal of the MthK channel as a template to model the open spHCN channel. Since this model failed to explain experimental results with cysteine-scanning and Cd^2+^ block experiments [3], [33], an additional constraint was applied by the authors to reorient the C-terminus of the S6 helix which reduced the bending of helix S6 of about 18° relative to MthK. More recently the open form of the KcsA channel has been published [13], and we took advantage of this knowledge to model the open form of hHCN4. In our model we did not impose any additional rotational constraint, and as a result the orientation of the C-terminal part of the helix S6 in hHCN4 is about 10° less kinked (at G502) than the MthK S6, a bending intermediate between those of the spHCN model and the MthK crystal structure.

### Conclusions

Our data indicate that the ivabradine binding site is located within the inner cavity of HCN4 channels, where the bound ivabradine is stabilized by several van der Waals and hydrophobic interactions. Although this happens both in the closed and in the open channel forms (Fig. 5), the drug binding mode differs substantially in the two channel states. In particular, the position acquired by the ivabradine N-atom in the closed channel is sufficiently close to the lowermost permeating ion binding site to antagonize their binding, in agreement with current-dependent block. According to our results, the major determinant of ivabradine binding to the channel is the structural integrity of the floor of the cavity in the closed channel, represented by the double crown built by residues Y506 and I510 in the tetrameric assembly.

Our data indicate that the affinity of ivabradine binding to the blocking site is higher in the closed than in the open state of the channel. This feature does not contrast with the property of "open channel block", since it simply reflects the need of channels to be open for drug molecules to reach their binding site. In fact, this functional property corresponds to our model predictions whereby the drug cannot reach, nor leave, the docking site when the channel is in the closed state.

Thus, the block mechanism can be summarized as follows: 1) ivabradine needs an open channel to access its binding site; 2) open channels, depolarization, and outward current flow favour access to the blocking site by means of a "current-dependent" mechanism; 3) when the channel is closed, the drug molecule is "trapped" in the cavity and cannot be displaced by either inward or outward flow; trapping depends strongly upon side chains of the Y506 and I510 residues.

Trapping of blocking molecules has been described already for the binding of ZD7288 to mHCN2 channels [24]. Although the mechanisms of block of ZD7288 on mHCN2 and of ivabradine on hHCN4 are clearly not identical, some specific similarities exist. For example, similar effects are observed when the structurally homologous residues I510 (hHCN4) and I432 (mHCN2) are replaced by alanine. On the other hand, also important differences exist. For example, Y506 (hHCN4) is involved in determining the steady-state hHCN4 block by ivabradine, but the corresponding mHCN2 Y428 has no effect on steady-state mHCN2 block by ZD7288. Overall these data suggest that while the blocking sites of both drugs reside in the channel cavity, the detailed molecular interactions with surrounding residues differ substantially in hHCN4 and mHCN2. This agrees with the notion that ZD7288 block is not current-dependent [10].

## Supporting Information

Figure S1 Membrane expression of L477A mutant channels. Videoconfocal images of HEK293 cells transfected with hHCN4 WT (top panels) and L477A mutant cDNA (bottom panels), and immunolabelled with anti hHCN4 antibodies (red). In both cases a strong membrane-associated fluorescence is detected, indicating that the protein localizes to the membrane. The lack of current expression in the L477A mutant channel (see Table S1) may therefore indicate that substitution of L477 functionally impairs the ability of the channel to carry the current. Each image represents the scanning of a single video-confocal section. Nuclei labeled with DAPI.
(TIF)

Figure S2 The Y506 side chain in F509A is rotated relative to wild-type channels. View of the interior of the hHCN4 wt (gray) and F509A mutant channels (orange) in the closed form. Side-chains of Y506 and F509 in the wt, and of Y506 and A509 in the mutant F509A channels are shown as ball-and-stick. In magenta is the best pose of docked ivabradine for the F509A mutant.
(TIF)

Materials and Methods S1 Methods for immunolabeling.
(DOC)

Table S1 Biophysical properties of hHCN4 WT and mutant channels expressed in HEK293 cells. V_1/2_, voltage of half-maximal activation; s, slope factor of activation curve; τ_act_, activation time constant measured at −140 mV (single exponential fit); τ_deact_, deactivation time constant measured at +5 mV (single exponential fit); *P<0.05 *vs* WT channels.
(DOC)

## References

[1] Biel M, Wahl-Schott C, Michalakis S, Zong X (2009) Hyperpolarization-activated cation channels: from genes to function. Physiol Rev 89: 847–885. 19584315 10.1152/physrev.00029.2008

[2] Shin KS, Rothberg BS, Yellen G (2001) Blocker state dependence and trapping in hyperpolarization-activated cation channels: evidence for an intracellular activation gate. J Gen Physiol 117: 91–101. 11158163 10.1085/jgp.117.2.91 PMC2217248

[3] Rothberg BS, Shin KS, Phale PS, Yellen G (2002) Voltage-controlled gating at the intracellular entrance to a hyperpolarization-activated cation channel. J Gen Physiol 119: 83–91. 11773240 10.1085/jgp.119.1.83 PMC2233860

[4] Giorgetti A, Carloni P, Mistrik P, Torre V (2005) A homology model of the pore region of HCN channels. Biophys J 89: 932–944. 15951376 10.1529/biophysj.104.045286 PMC1366642

[5] Stansfeld PJ, Gedeck P, Gosling M, Cox B, Mitcheson JS, et al (2007) Drug block of the hERG potassium channel: insight from modeling. Proteins 68: 568–580. 17444521 10.1002/prot.21400

[6] Long SB, Campbell EB, MacKinnon R (2005) Crystal structure of a mammalian voltage-dependent Shaker family K^+^ channel. Science 309: 897–903. 16002581 10.1126/science.1116269

[7] Robinson RB, Siegelbaum SA (2003) Hyperpolarization-activated cation currents: from molecules to physiological function. Annu Rev Physiol 65: 453–480. 12471170 10.1146/annurev.physiol.65.092101.142734

[8] Postea O, Biel M (2011) Exploring HCN channels as novel drug targets. Nat Rev Drug Discov 10: 903–914. 22094868 10.1038/nrd3576

[9] Bois P, Bescond J, Renaudon B, Lenfant J (1996) Mode of action of bradycardic agent, S 16257, on ionic currents of rabbit sinoatrial node cells. Br J Pharmacol 118(4) 1051–1057. 10.1111/j.1476-5381.1996.tb15505.x PMC1909508 8799581

[10] Bucchi A, Baruscotti M, DiFrancesco D (2002) Current-dependent block of rabbit sino-atrial node I_f_ channels by ivabradine. J Gen Physiol 120: 1–13. 12084770 10.1085/jgp.20028593 PMC2238187

[11] Bucchi A, Tognati A, Milanesi R, Baruscotti M, DiFrancesco D (2006) Properties of ivabradine-induced block of HCN1 and HCN4 pacemaker channels. J Physiol 572: 335–346. 16484306 10.1113/jphysiol.2005.100776 PMC1779671

[12] Doyle DA, Morais CJ, Pfuetzner RA, Kuo A, Gulbis JM, et al (1998) The structure of the potassium channel: molecular basis of K^+^ conduction and selectivity. Science 280: 69–77. 9525859 10.1126/science.280.5360.69

[13] Cuello LG, Jogini V, Cortes DM, Pan AC, Gagnon DG, et al (2010) Structural basis for the coupling between activation and inactivation gates in K^+^ channels. Nature 466: 272–275. 20613845 10.1038/nature09136 PMC3033755

[14] Claros MG, von HG (1994) TopPred II: an improved software for membrane protein structure predictions. Comput Appl Biosci 10: 685–686. 7704669 10.1093/bioinformatics/10.6.685

[15] Krogh A, Larsson B, von HG, Sonnhammer EL (2001) Predicting transmembrane protein topology with a hidden Markov model: application to complete genomes. J Mol Biol 305: 567–580. 11152613 10.1006/jmbi.2000.4315

[16] Tusnady GE, Simon I (2001) The HMMTOP transmembrane topology prediction server. Bioinformatics 17: 849–850. 11590105 10.1093/bioinformatics/17.9.849

[17] Thompson JD, Higgins DG, Gibson TJ (1994) CLUSTAL W: improving the sensitivity of progressive multiple sequence alignment through sequence weighting, position-specific gap penalties and weight matrix choice. Nucleic Acids Res 22: 4673–4680. 7984417 10.1093/nar/22.22.4673 PMC308517

[18] Sali A, Blundell TL (1993) Comparative protein modelling by satisfaction of spatial restraints. J Mol Biol 234: 779–815. 8254673 10.1006/jmbi.1993.1626

[19] Shen MY, Sali A (2006) Statistical potential for assessment and prediction of protein structures. Protein Sci 15: 2507–2524. 17075131 10.1110/ps.062416606 PMC2242414

[20] Murshudov GN, Vagin AA, Dodson EJ (1997) Refinement of macromolecular structures by the maximum-likelihood method. Acta Crystallogr D Biol Crystallogr 53: 240–255. 15299926 10.1107/S0907444996012255

[21] Laskowski RA, Moss DS, Thornton JM (1993) Main-chain bond lengths and bond angles in protein structures. J Mol Biol 231: 1049–1067. 8515464 10.1006/jmbi.1993.1351

[22] Altomare C, Bucchi A, Camatini E, Baruscotti M, Viscomi C, et al (2001) Integrated allosteric model of voltage gating of HCN channels. J Gen Physiol 117: 519–532. 11382803 10.1085/jgp.117.6.519 PMC2232403

[23] Morris GM, Goosell DS, Halliday RS, Huey R, Hart WE, et al (2012) Automated Docking Using a Lamarckian Genetic Algorithm and and Empirical Binding Free Energy Function. J Comput Chem 19: 1639–1662.

[24] Cheng L, Kinard K, Rajamani R, Sanguinetti MC (2007) Molecular mapping of the binding site for a blocker of hyperpolarization-activated, cyclic nucleotide-modulated pacemaker channels. J Pharmacol Exp Ther 322: 931–939. 17578902 10.1124/jpet.107.121467

[25] Chan YC, Wang K, Au KW, Lau CP, Tse HF, et al (2009) Probing the bradycardic drug binding receptor of HCN-encoded pacemaker channels. Pflugers Arch 459: 25–38. 19756722 10.1007/s00424-009-0719-2 PMC2765624

[26] Thollon C, Vilaine JP (2010) I_f_ inhibition in cardiovascular diseases. Adv Pharmacol 59: 53–92. 20933199 10.1016/S1054-3589(10)59003-3

[27] DiFrancesco JC, Barbuti A, Milanesi R, Coco S, Bucchi A, et al (2011) Recessive loss-of-function mutation in the pacemaker HCN2 channel causing increased neuronal excitability in a patient with idiopathic generalized epilepsy. J Neurosci 31: 17327–17337. 22131395 10.1523/JNEUROSCI.3727-11.2011 PMC6623833

[28] Emery EC, Young GT, Berrocoso EM, Chen L, McNaughton PA (2011) HCN2 ion channels play a central role in inflammatory and neuropathic pain. Science 333: 1462–1466. 21903816 10.1126/science.1206243

[29] Fernandez D, Ghanta A, Kauffman GW, Sanguinetti MC (2004) Physicochemical features of the HERG channel drug binding site. J Biol Chem 279: 10120–10127. 14699101 10.1074/jbc.M310683200

[30] Mitcheson JS, Chen J, Lin M, Culberson C, Sanguinetti MC (2000) A structural basis for drug-induced long QT syndrome. Proc Natl Acad Sci USA 97: 12329–12333. 11005845 10.1073/pnas.210244497 PMC17341

[31] Sanchez-Chapula JA, Ferrer T, Navarro-Polanco RA, Sanguinetti MC (2003) Voltage-dependent profile of human ether-a-go-go-related gene channel block is influenced by a single residue in the S6 transmembrane domain. Mol Pharmacol 63: 1051–1058. 12695533 10.1124/mol.63.5.1051

[32] Roncaglia P, Mistrik P, Torre V (2002) Pore topology of the hyperpolarization-activated cyclic nucleotide-gated channel from sea urchin sperm. Biophys J 83: 1953–1964. 12324414 10.1016/S0006-3495(02)73957-X PMC1302285

[33] Rothberg BS, Shin KS, Yellen G (2003) Movements near the gate of a hyperpolarization-activated cation channel. J Gen Physiol 122: 501–510. 14557404 10.1085/jgp.200308928 PMC2229576

