# Supplementary figures and images for: Identification of the Molecular Site of Ivabradine Binding to HCN4 Channels
Source: PLoS One. 2013 Jan 4;8(1):e53132. doi: 10.1371/journal.pone.0053132 (PMC3537762; doi:10.1371/journal.pone.0053132)

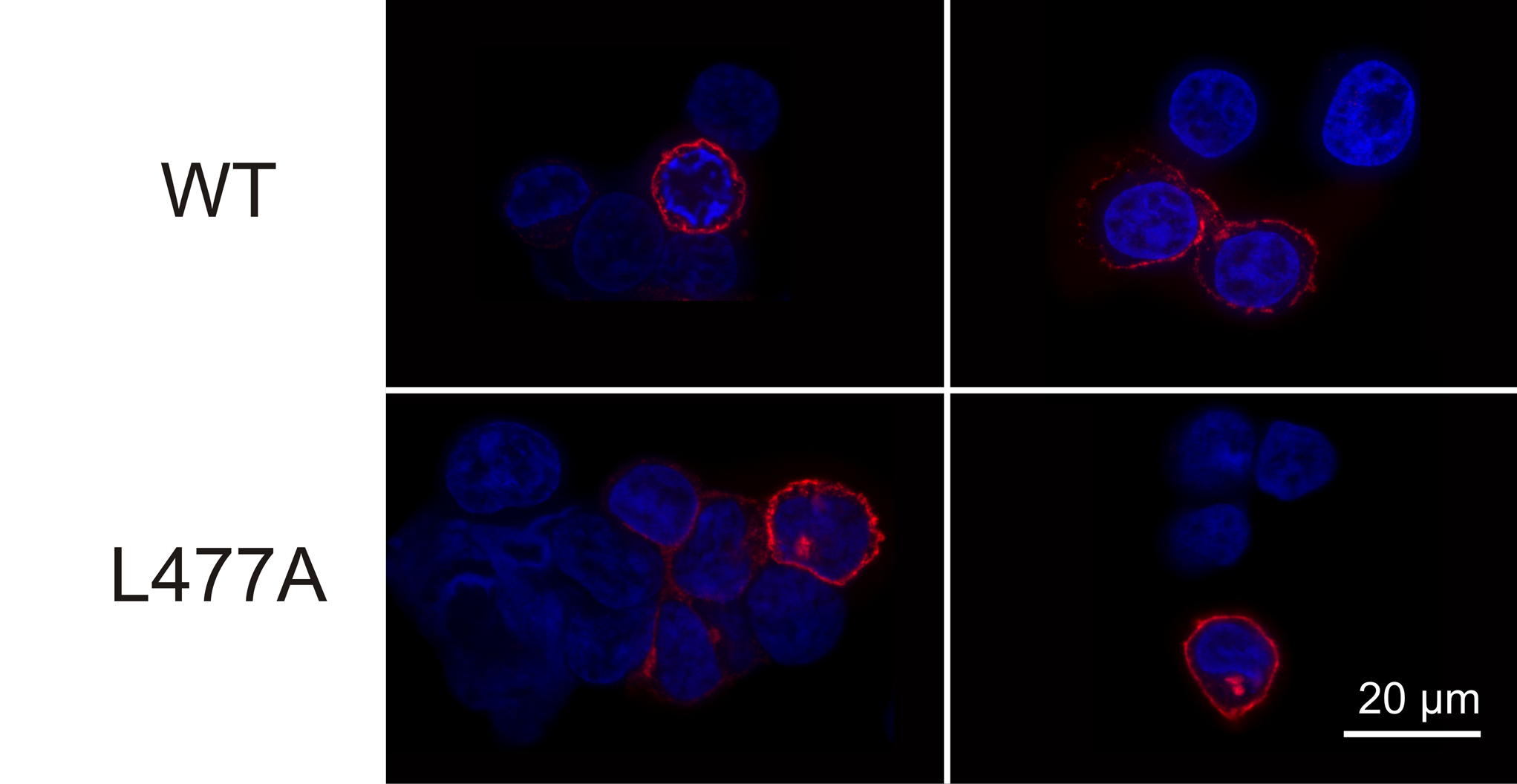

Supplement: Figure S1 — Membrane expression of L477A mutant channels. Videoconfocal images of HEK293 cells transfected with hHCN4 WT (top panels) and L477A mutant cDNA (bottom panels), and immunolabelled with anti hHCN4 antibodies (red). In both cases a strong membrane-associated fluorescence is detected, indicating that the protein localizes to the membrane. The lack of current expression in the L477A mutant channel (see Table S1) may therefore indicate that substitution of L477 functionally impairs the ability of the channel to carry the current. Each image represents the scanning of a single video-confocal section. Nuclei labeled with DAPI. (TIF) [file pone.0053132.s001.tif]

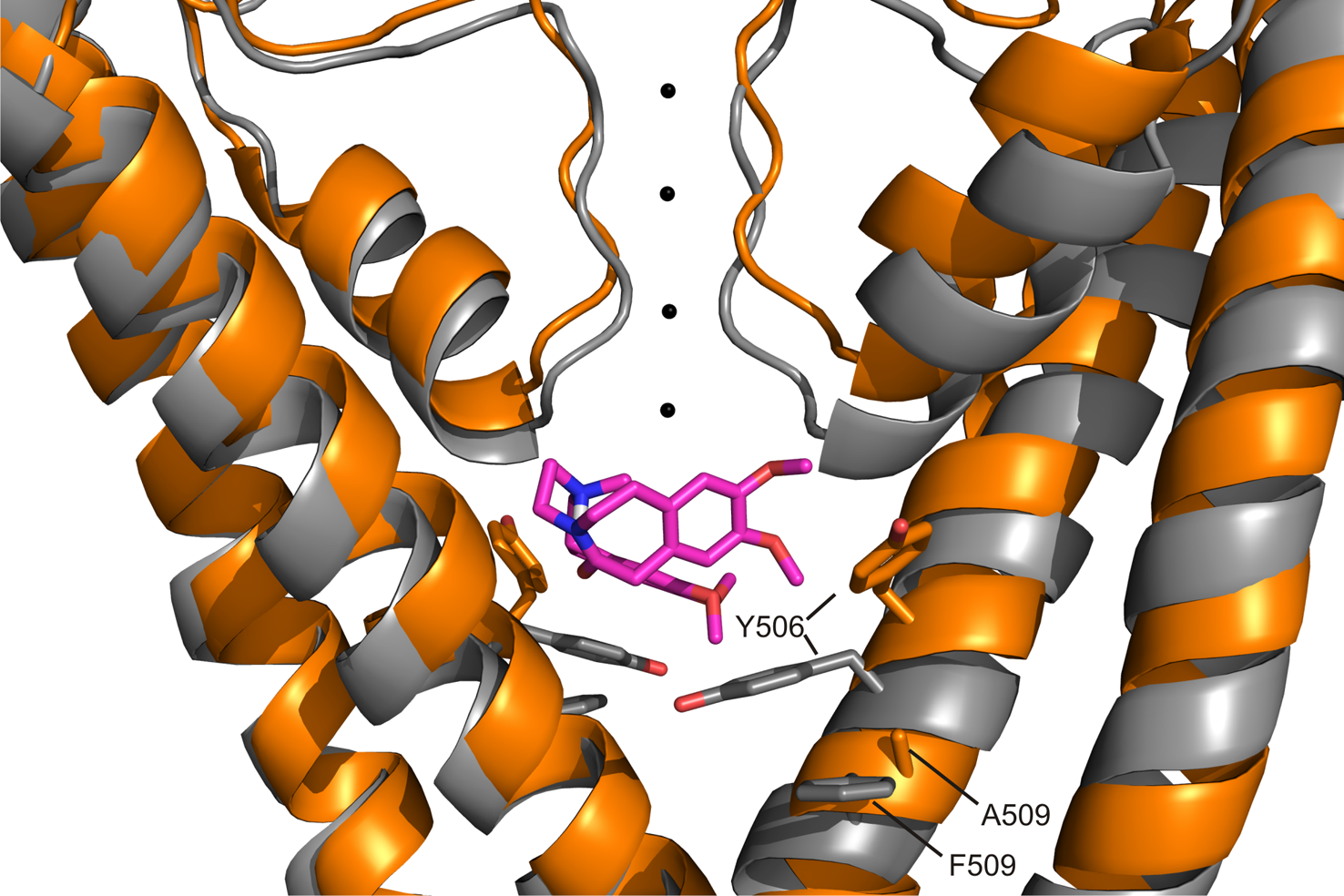

Supplement: Figure S2 — The Y506 side chain in F509A is rotated relative to wild-type channels. View of the interior of the hHCN4 wt (gray) and F509A mutant channels (orange) in the closed form. Side-chains of Y506 and F509 in the wt, and of Y506 and A509 in the mutant F509A channels are shown as ball-and-stick. In magenta is the best pose of docked ivabradine for the F509A mutant. (TIF) [file pone.0053132.s002.tif]
