# Supplementary material for: Identification of the Molecular Site of Ivabradine Binding to HCN4 Channels
Source: PLoS One. 2013 Jan 4;8(1):e53132. doi: 10.1371/journal.pone.0053132 (PMC3537762; doi:10.1371/journal.pone.0053132)
Supplement: Materials and Methods S1 — Methods for immunolabeling. (DOC) [file pone.0053132.s003.doc]

SUPPLEMENTAL INFORMATION

**Identification of the molecular site of ivabradine binding to HCN4 channels**

Annalisa Bucchi*^1,2^, Mirko Baruscotti*^1,2^, Marco Nardini^3^, Andrea Barbuti^1,2^, Stefano Micheloni^1^, Martino Bolognesi^3^ and Dario DiFrancesco^$1,2^

^1^The PaceLab and ^3^Laboratory of Protein Biochemistry, Department of Life Sciences, Università degli Studi di Milano, via Celoria 26, 20133 Milano, Italy.

^2^Centro Interuniversitario di Medicina Molecolare e Biofisica Applicata, Università degli Studi di Milano, via Celoria 26, 20133 Milano, Italy.

Short title: Molecular model of HCN4 block by ivabradine

* These authors contributed equally to this work

^$^ Corresponding author

**SI MATERIALS AND METHODS**

Immunolabelling

HEK cells transiently transfected with 1.0 μg of either hHCN4 or hHCN4L477A plasmids were plated on polysine glass slides and fixed in 4% paraformaldehyde (8 min at 4 °C). Cells were rinsed with PBS containing 0.1 M glycine (20 min), and incubated (30 min) with PBS solution containing 0.3% Triton X-100, 1% BSA, and 10% donkey serum (Sigma) to induce permeabilization and block. Primary and secondary antibodies were diluted in PBS solution. Incubation (1:100) with the primary anti-HCN4 antibody (Alomone Labs) was carried out overnight at 4 °C. Cells were then thoroughly rinsed in PBS solution before exposure (1 h at RT) to the secondary antibody (1:1000 of AlexaFluor 594 donkey anti-rabbit; Invitrogen). Finally, cells were washed for 30 min in PBS solution before mounting with Vectashield mounting medium with DAPI (Vector Laboratories). Videoconfocal images of WT and L477A transfected cells are shown in Figure S1.
