# Supplementary material for: Identification of the Molecular Site of Ivabradine Binding to HCN4 Channels
Source: PLoS One. 2013 Jan 4;8(1):e53132. doi: 10.1371/journal.pone.0053132 (PMC3537762; doi:10.1371/journal.pone.0053132)
Supplement: Table S1 — Biophysical properties of hHCN4 WT and mutant channels expressed in HEK293 cells. V1/2, voltage of half-maximal activation; s, slope factor of activation curve; τact, activation time constant measured at −140 mV (single exponential fit); τdeact, deactivation time constant measured at +5 mV (single exponential fit); *P<0.05 vs WT channels. (DOC) [file pone.0053132.s004.doc]

Table S1. Biophysical properties of hHCN4 WT and mutant channels expressed in HEK293 cells.

| **Channel** | **V_1/2_**  **(mV)** | **s**  **(mV)** | **n** | **τ_act_  (ms)**  **@ -140 mV** | **n** | **τ_deact_ (ms)**  **@ +5 mV** | **n** |
| --- | --- | --- | --- | --- | --- | --- | --- |
| **WT** | -76.8±2.4 | 9.6±0.4 | 6 | 81.1±5.0 | 22 | 73.6±3.5 | 14 |
| **L477A** | no functional expression | | | | | | 52 |
| **C478A** | -79.4±2.1 | 7.8±0.9 | 7 | 76.5±4.3 | 33 | 64.8±3.2 | 11 |
| **A503V** | -94.5±2.4 * | 12.4±0.6 * | 7 | 128.7±6.3 * | 15 | 24.8±4.7* | 6 |
| **Y506A** | -94.8±1.6 * | 10.5±1.7 | 6 | 187.6±18.9 * | 15 | 45.5±5.8* | 6 |
| **A507V** | -88.4±2.8 * | 11.8±1.0 | 5 | 153.4±7.5* | 28 | 32.4±2.9* | 15 |
| **M508A** | -78.9±1.8 | 10.0±0.6 | 6 | 98.4±5.5* | 23 | 57.0±2.7* | 7 |
| **F509A** | -78.1±0.9 | 12.6±0.8* | 5 | 187.5±10.4* | 25 | 464.4±37.4* | 12 |
| **I510A** | -73.9±3.6 | 11.1±0.8 | 5 | 21.6±1.7* | 11 | 53.4±5.0* | 6 |
| **Y506A-I510A** | -77.3±2.1 | 11.2±1.3 | 5 | 79.1±6.5 | 15 | 50.7±6.5* | 4 |
| **Y506A-F509A** | -84.6±2.1* | 8.7±0.6 | 4 | 161.8±7.6* | 14 | 70.1±7.8 | 8 |
| **Y506A-F509A-I510A** | -82.4±2.4 | 11.2±0.1* | 4 | 99.6±6.1* | 11 | 41.1±5.9* | 7 |
